# Polyunsaturated Fatty Acids as Modulators of K_V_7 Channels

**DOI:** 10.3389/fphys.2020.00641

**Published:** 2020-06-11

**Authors:** Johan E. Larsson, Damon J. A. Frampton, Sara I. Liin

**Affiliations:** Department of Biomedical and Clinical Sciences, Linköping University, Linköping, Sweden

**Keywords:** docosahexaenoic acid, KCNE, KCNQ, K_V_7, lipid, polyunsaturated fatty acid, voltage-gated potassium channel

## Abstract

Voltage-gated potassium channels of the K_V_7 family are expressed in many tissues. The physiological importance of K_V_7 channels is evident from specific forms of disorders linked to dysfunctional K_V_7 channels, including variants of epilepsy, cardiac arrhythmia and hearing impairment. Thus, understanding how K_V_7 channels are regulated in the body is of great interest. This Mini Review focuses on the effects of polyunsaturated fatty acids (PUFAs) on K_V_7 channel activity and possible underlying mechanisms of action. By summarizing reported effects of PUFAs on K_V_7 channels and native K_V_7-mediated currents, we conclude that the generally observed effect is a PUFA-induced increase in current amplitude. The increase in current is commonly associated with a shift in the voltage-dependence of channel opening and in some cases with increased maximum conductance. Auxiliary KCNE subunits, which associate with K_V_7 channels in certain tissues, may influence PUFA effects, though findings are conflicting. Both direct and indirect activating PUFA effects have been described, direct effects having been most extensively studied on K_V_7.1. The negative charge of the PUFA head-group has been identified as critical for electrostatic interaction with conserved positively charged amino acids in transmembrane segments 4 and 6. Additionally, the localization of double bonds in the PUFA tail tunes the apparent affinity of PUFAs to K_V_7.1. Indirect effects include those mediated by PUFA metabolites. Indirect inhibitory effects involve K_V_7 channel degradation and re-distribution from lipid rafts. Understanding how PUFAs regulate K_V_7 channels may provide insight into physiological regulation of K_V_7 channels and bring forth new therapeutic strategies.

## Introduction

It is well established that lipids can influence the function of voltage-gated ion channels and their organization in the membrane (Dart, 2010; Elinder and Liin, 2017). Specific members within the K_V_7 family of voltage-gated potassium channels have been under intense study, owing to their physiological regulation by the phospholipid PIP_2_ (phosphatidylinositol 4,5-bisphosphate; reviewed in Zaydman and Cui, 2014; Taylor and Sanders, 2017). However, several years of studies have revealed that unesterified, so called free fatty acids, may also regulate K_V_7 channel function. Polyunsaturated fatty acids (PUFAs) in particular have emerged as interesting K_V_7 modulators. This Mini Review will provide a brief essential background on K_V_7 channels and PUFAs, followed by a summary of our present understanding of PUFAs as K_V_7 channel modulators and potential future developments.

## Physiological Role and General Architecture of K_V_7 Channels

**FIGURE 1:**
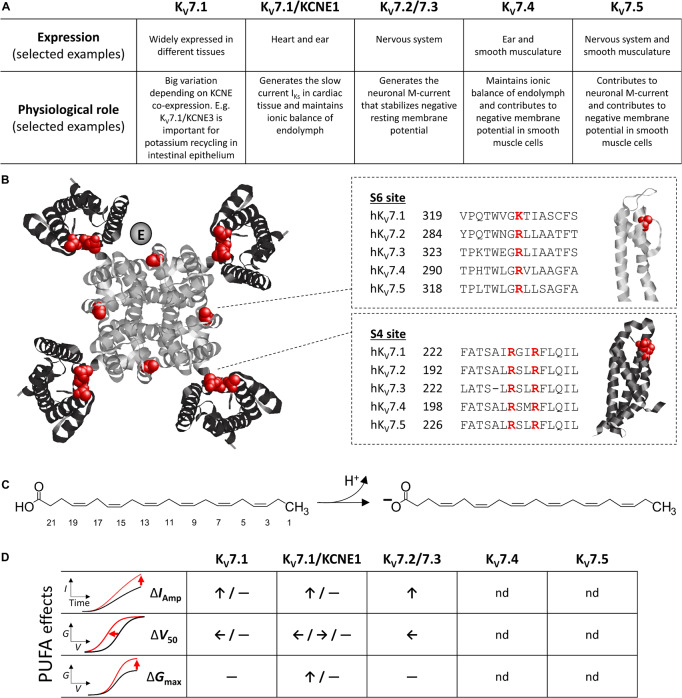
Overview of K_V_7 channel and PUFA molecular structures, and typified PUFA effects. **(A)** Schematic overview of K_V_7 subtype expression and functional role. Note that these are examples, as some K_V_7 subtypes have widespread expression and function. **(B)** Top view of K_V_7.1 (PDB: 5VMS) with central pore domain in gray and peripheral voltage-sensing domains in black. Putative localization of KCNE at one K_V_7.1 channel subunit is indicated [each subunit may accommodate one KCNE subunit (Sun and MacKinnon, 2020)]. Experimentally identified positively charged residues important for PUFA effects in K_V_7.1 (Liin et al., 2018) are highlighted in red. Sequence alignment of the S6 site (important for *G*_max_ effect) and S4 site (important for *V*_50_ effect) of all K_V_7 isoforms are provided along with a side view of relevant channel domain. **(C)** Structure of the PUFA DHA, which has a carboxyl head linked to a 22-carbon long aliphatic tail with six *cis* double bonds. Deprotonation of the carboxyl head occurs at pH exceeding the pKa of the head-group, this endows DHA with a single negative charge. **(D)** Schematic overview of PUFA effect on current amplitude (*I*_A__mp_), mid-point of the *G*(*V*) curve (*V*_50_), and maximum conductance (*G*_max_) on indicated K_V_7 subtypes. Please refer to Table 1 for further details. nd denotes not determined.

The K_V_7 family of voltage-gated potassium channels, of which there are five different isoforms, termed K_V_7.1 to K_V_7.5, are encoded by the *KCNQ* genes. The tissue distribution of K_V_7 subtypes varies, and the channels serve different physiological roles (Figure 1A). K_V_7.1 (in complex with the auxiliary KCNE1 protein, see further details below) is most famous for generating the slow current I_Ks_ in cardiomyocytes, important for cardiac repolarization (Barhanin et al., 1996; Sanguinetti et al., 1996), while also maintaining the ionic balance of endolymph in the inner ear (Neyroud et al., 1997). Heteromers of K_V_7.2 and K_V_7.3 generate the neuronal M-current (I_M_) important for stabilizing the negative resting membrane potential of neurons and thereby regulating excitability (Wang et al., 1998; Cooper et al., 2000). K_V_7.4 also contributes to maintaining the ionic balance of endolymph (Kubisch et al., 1999; Kharkovets et al., 2000), as well as contributing to the negative membrane potential in smooth muscle cells at rest (Jepps et al., 2011; Chadha et al., 2014; Jepps et al., 2015). K_V_7.5 is suggested to form heteromers with other neuronal and smooth muscle K_V_7 subtypes and contribute to their function in these tissues (Lerche et al., 2000; Chadha et al., 2014). Because of their important role in physiology, dysfunctional K_V_7 channels are often linked to disorders characterized by abnormal potassium ion conductance, including cardiac arrhythmia, hearing impairment, epilepsy, pain, and hypertension (Barrese et al., 2018). For a more extensive overview of expression and physiological and pathological implications of K_V_7 channels, we recommend recent reviews on this topic (Barrese et al., 2018; Miceli et al., 2018).

Each *KCNQ* gene encodes one K_V_7 subunit, composed of six transmembrane segments (helices, referred to as S1–S6) and intracellular *N* and *C* termini (Jentsch, 2000). Four such K_V_7 subunits assemble into functional tetrameric K_V_7 channels, which can be either homomeric or heteromeric (Schwake et al., 2003; Schwake et al., 2006). A cryogenic electron microscopy structure of K_V_7.1 visualizes how transmembrane segments S5–S6 of all four subunits form the central pore domain, whereas S1 to S4 of each subunit form the peripheral voltage-sensing domains (Figure 1B; Sun and MacKinnon, 2017). The determined structure reveals a domain swapped architecture, meaning that the voltage-sensing domain of one subunit lies adjacent to the pore-forming segments of its neighboring subunit (Sun and MacKinnon, 2017). Conserved, positively charged arginines in S4 of the voltage-sensing domain act as the primary voltage-sensing residues in K_V_7 channels (Panaghie and Abbott, 2007; Miceli et al., 2008). Under hyperpolarized conditions, S4 is in a “downwards,” internal, conformation, and the gate (located in the internal part of S6) of the central ion conducting pore is closed (Cui, 2016). Upon depolarization, S4 moves via intermediate conformations to an “upwards,” external, conformation (Barro-Soria et al., 2014; Zaydman et al., 2014; Taylor et al., 2020), which triggers opening of the S6 gate and potassium conductance through the pore (Cui, 2016; Hou et al., 2020). Endogenous or exogenous ligands and auxiliary proteins may interact with and modulate the activity of K_V_7 channels by altering S4 movement, gate opening, or the coupling between the two. For instance, auxiliary KCNE subunits (KCNE1-5) interact with K_V_7 channels to modulate their expression and biophysical properties (Abbott, 2016; Barrese et al., 2018). Several studies assign the single-transmembrane segment of KCNE subunits to a space between neighboring voltage-sensing domains (see Figure 1B for putative KCNE localization; e.g., Chung et al., 2009; Xu et al., 2013; Sun and MacKinnon, 2020).

## Structure and Properties of PUFAs

Polyunsaturated fatty acids are naturally occurring lipids known to modulate the activity of numerous voltage-gated ion channels (reviewed in Elinder and Liin, 2017). PUFA characteristics include a carboxylic acid “head-group” and an unbranched, aliphatic hydrocarbon “tail” with at least two double bonds in *cis* geometry (Figure 1C). The carboxyl head-group is either uncharged or negatively charged, depending on the protonation status (Figure 1C). The pKa value of carboxyl heads of PUFAs in proximity of ion channels approaches 7.4 (Hamilton, 1998; Liin et al., 2015), and so approximately 50% of PUFA head-groups are expected to be deprotonated and negatively charged at physiological pH. The *cis* double bonds allow polyunsaturated tails to bend and explore geometries that are not possible in the absence of *cis* double bonds (Feller, 2008; Yazdi et al., 2016). PUFAs are typically described according to the number of carbons and double bonds in the tail or their omega classification. Figure 1C shows the structure of docosahexaenoic acid (DHA), which has 22 carbons and 6 double bonds in its tail (22:6) and is an omega-3 PUFA (i.e., the first double bond is at the 3rd carbon from the methyl end). Examples of other physiologically relevant PUFAs that can be obtained via diet or synthesized from other essential fatty acids in the human body are arachidonic acid (AA, 20:4, omega-6), linoleic acid (LA, 18:2, omega-6), alpha-linolenic acid (ALA, 18:3, omega-3), and eicosapentaenoic acid (EPA, 20:5, omega-3). Reported physiological levels of PUFAs in plasma, serum, or cerebrospinal fluid are around 10–50 μM, but may reach higher levels during, for instance, excessive dietary PUFA intake (Conquer and Holub, 1998; Fraser et al., 2003; Brouwer et al., 2006).

## Reported PUFA Effects on K_V_7 Channels and Currents

Table 1 summarizes reported PUFA effects on heterologously expressed K_V_7 channels and isolated native currents generated by K_V_7 channels. Notably, PUFA effects have thus far only been studied on K_V_7.1–K_V_7.3. A majority of studies have explored the acute effects of extracellular application of AA, ALA, DHA, EPA, or LA. These effects are achieved within the range of minutes after PUFA application. The most consistently observed PUFA effect on heterologously expressed K_V_7 channels is an increased current amplitude at a range of negative voltages, an effect reported for several PUFAs tested on K_V_7.1 and heteromeric K_V_7.2/7.3 channels (Table 1). Several studies report a PUFA-induced shift in the conductance *versus* voltage [*G*(*V*)] curve toward more negative voltages (described as negative shift in *V*_50_ in Table 1; Liin et al., 2015; Moreno et al., 2015; Liin et al., 2016a). Additionally, PUFAs may increase the maximum conductance observed at the most positive voltages (described as increase in *G*_max_ in Table 1; Bohannon et al., 2020). Both the shift in *V*_50_ and increase in *G*_max_ may contribute to the overall increase in current amplitude at different voltages. For clarity, we will refer to the three effects as “increase in current amplitude,” “shift in *V*_50_,” and “increase in *G*_max_.” Figure 1D schematically illustrates PUFA effects on each of these parameters on different K_V_7 subtypes, whereas Table 1 reflects the complexity of effects and highlights examples of reported effects at specific PUFA concentrations. Typically, PUFA concentrations of 7 μM or higher are required to induce significant effects. Note that PUFA effects on current amplitude have typically been quantified at relatively depolarized voltages, which may not be physiologically relevant. However, because PUFAs generally also shift *V*_50_ toward negative voltages, the relative increase in current amplitude is larger at less depolarized voltages. Although studies rarely quantify PUFA effects in a more physiologically relevant voltage range, a prominent increase in current amplitude at less depolarized voltages is observed in several studies (Doolan et al., 2002; Liin et al., 2015; Moreno et al., 2015; Liin et al., 2016a).

**TABLE 1:** Summary of reported PUFA effects on indicated K_V_7 channels/currents.

Channel/current	Experimental system	PUFA	Time perspective	Main effect(s) reported^†^	References
**Heterologous expression:**					
hK_V_7.1	*Xenopus* oocytes	DHA, EPA	Acute	Faster activation kinetics ∼*5% at* + *100 mV by 20* μ*M EPA*	Doolan et al., 2002
hK_V_7.1	*Xenopus* oocytes	DHA, EPA	Acute	Increased current amplitude Negative shift in *V*_50_ *-9 to -14 mV by 70* μ*M DHA or EPA*	Liin et al., 2015
hK_V_7.1	COS7	EPA	Acute	(No effect by 20 μM EPA)	Moreno et al., 2015
hK_V_7.1/KCNE1	*Xenopus* oocytes	DHA, EPA	Acute	Increased current amplitude *29% at* + *40 mV by 20* μ*M DHA* Slowed activation kinetics ∼*73% at* + *100 mV by 20* μ*M EPA*	Doolan et al., 2002
hK_V_7.1/KCNE1	COS7	DHA, EPA	Acute	Increased current amplitude *83% at* + *60 mV by 20* μ*M DHA* *37% at* + *60 mV by 20* μ*M EPA* Negative shift in *V*_50_ *-9 mV by 20* μ*M EPA*	Moreno et al., 2015
hK_V_7.1/KCNE1	COS7	DHA, EPA	Prolonged	Negative or positive shift in *V*_50_ *-8 mV by 20* μ*M DHA* + *10 mV by 20* μ*M EPA*	Moreno et al., 2015
hK_V_7.1/KCNE1	*Xenopus* oocytes	DHA	Acute	(No effect by 70 μM DHA)	Liin et al., 2015
hK_V_7.1/KCNE1	*Xenopus* oocytes	LA, DHA	Acute	Increased current amplitude and *G*_max_ ∼*70% in G_max_ by 20* μ*M DHA*	Bohannon et al., 2020
hK_V_7.2/7.3	*Xenopus* oocytes	ALA, DHA, EPA	Acute	Increased current amplitude Negative shift in *V*_50_ *-7 to -11 mV by 70* μ*M ALA, DHA, or EPA*	Liin et al., 2016a
hK_V_7.2/7.3/KCNE1	*Xenopus* oocytes	DHA	Acute	Increased current amplitude Negative shift in *V*_50_ *-8 mV by 70* μ*M DHA*	Liin et al., 2016a
hK_V_7.2/7.3/KCNE2	*Xenopus* oocytes	DHA	Acute	Increased current amplitude Negative shift in *V*_50_ *-5 mV by 70* μ*M DHA*	Liin et al., 2016a
**Native current:**					
I_Ks_	Guinea pig ventricular cardiomyocytes	DHA	Acute	Increased current amplitude *38% at* + *20 mV by 10* μ*M DHA* Accelerated deactivation *19% at -40 mV by 10* μ*M DHA*	Moreno et al., 2015
I_M_	Rodent neuroblastoma cells	AA, DHA	Acute	Biphasic effects (both decreased and increased current amplitude) *observed for 5–50* μ*M PUFA*	Behe et al., 1992
I_M_	Bullfrog sympathetic neurons	AA	Acute	Increased current amplitude ∼*30–60% by 5–100* μ*M AA*	Villarroel, 1993, 1994; Yu, 1995

*^†^Effects may vary between tested PUFAs (in such cases, most prominent effects have been listed and quantitative examples are provided in italic). AA denotes arachidonic acid, ALA denotes linolenic acid, LA denotes linoleic acid, DHA denotes docosahexaenoic acid, EPA denotes eicosapentaenoic acid. Acute effects indicate effects observed within minutes. Prolonged effects indicate effects observed within hours to days. *V*_*50*_ denotes the mid-point of the *G*(*V*) curve and is used to report on changes in the voltage dependence of channel opening. *G*_*max*_ denotes the top of the *G*(*V*) curve and is used to report on changes in the maximum conductance. COS7 is a fibroblast cell line derived from the African green monkey *C. aethiops*.*

In addition, PUFA effects on opening and closing kinetics have been described (Doolan et al., 2002; Moreno et al., 2015). However, effects on kinetics appear complex, with a range of described effects varying from PUFA to PUFA (Doolan et al., 2002; Moreno et al., 2015). Moreover, prolonged PUFA exposure over several days triggers a plethora of convoluted effects resulting in either negative *or* positive shift in *V*_50_, depending on PUFA (Moreno et al., 2015).

Reported PUFA effects on native K_V_7 currents largely agree with effects on heterologously expressed K_V_7 channels (Table 1). In a majority of guinea pig ventricular cardiomyocytes, acute application of DHA increases I_Ks_ amplitude (presumably generated by K_V_7.1/KCNE1 channels; Moreno et al., 2015). However, in a subset of cardiomyocytes DHA instead decreases I_Ks_ amplitude (Moreno et al., 2015). Several studies observe increases in I_M_ amplitude induced by AA or DHA in bullfrog sympathetic neurons (which presumably is generated by K_V_7.2/7.3 channels; Villarroel, 1993, 1994; Yu, 1995).

## Impact of KCNE Subunits on PUFA Effects

Because KCNE subunits associate with K_V_7 channels in multiple tissues, evaluating the impact of KCNE co-expression on PUFA effects is of interest. Auxiliary subunits have been shown to affect the response of other K_V_ channels to PUFAs. For instance, DHA, or AA augmentation of the Slo1 channel is potentiated or enabled, respectively, when the channel is co-expressed with specific β subunits (Sun et al., 2007; Hoshi et al., 2013a). Furthermore, AA modulation of K_V_4 channel inactivation kinetics is only observed if K_V_4.2 or K_V_4.3 are co-expressed with their KChIP subunit (Holmqvist et al., 2001). We found that neither KCNE1 nor KCNE2 co-expression with K_V_7.2/7.3 affected the ability of DHA to shift *V*_50_ of K_V_7.2/7.3 (Liin et al., 2016a). However, the occurrence and physiological relevance of complexes formed by K_V_7.2/7.3 and KCNE subunits remains questionable at present. Conflicting results are found regarding the impact of KCNE1 co-expression with K_V_7.1 on PUFA effects (Table 1 and Figure 1D). Doolan et al. (2002) report that KCNE1 is required for DHA-mediated effects on K_V_7.1 current amplitude, as they find that 20 μM of DHA increases the current amplitude of K_V_7.1/KCNE1, but not K_V_7.1. By comparison, 20 μM of EPA slowed activation kinetics for K_V_7.1/KCNE1 without affecting current amplitude (Doolan et al., 2002). In contrast, we report that DHA at concentrations of 7 μM or higher shifts *V*_50_ of K_V_7.1 toward negative voltages and that the presence of KCNE1 largely abolishes this *V*_50_ effect (Liin et al., 2015; Bohannon et al., 2020). However, we observe a DHA-induced increase in K_V_7.1/KCNE1 current amplitude and *G*_max_ (Bohannon et al., 2020). A third study, by Moreno and colleagues, reports that 20 μM of DHA or EPA increases K_V_7.1/KCNE1 current amplitude and shifts *V*_50_ of K_V_7.1/KCNE1 toward negative voltages (Moreno et al., 2015). However, no EPA effect is observed on K_V_7.1 alone (Moreno et al., 2015). The reason for these conflicting findings is not clear. One contributing factor may be the use of different expression systems (*Xenopus* oocytes *versus* mammalian COS7 cells). As previously remarked (Valenzuela, 2016), higher PUFA concentrations may be required in experiments with *Xenopus* oocytes than for mammalian cells to induce comparable effects. Another aspect to consider may be the method of PUFA application. We note larger DHA effects on current amplitude upon constant DHA perfusion compared to a single DHA application (Liin et al., 2015; Bohannon et al., 2020). Both Doolan *et al.*, and Moreno *et al.*, describe results employing PUFA perfusion.

## PUFA Properties Important for K_V_7 Effects

Previous studies have shown that PUFAs may interact directly with diverse ion channels to modulate their activity (Elinder and Liin, 2017). Moreno et al. (2015) concluded that the concurrent increase in K_V_7.1/KCNE1 current amplitude and negative shift in *V*_50_ implicate an effect on channel gating as the primary mechanism. We made the same observation in our initial work (Liin et al., 2015). In subsequent work we have since explored this possibility utilizing PUFA analogs, site-directed mutagenesis, and pH manipulation.

We find that a negatively charged PUFA head-group is critical to the shift in *V*_50_ of K_V_7.1 and K_V_7.2/7.3 toward negative voltages. Fully deprotonated PUFAs (promoted by an alkaline extracellular solution) cause larger negative *V*_50_ shifts (Liin et al., 2015; Larsson et al., 2018; Bohannon et al., 2019). Protonated PUFAs and uncharged PUFA analogs (PUFA methyl esters) are unable to shift *V*_50_ (Liin et al., 2015, 2016a). Positively charged PUFA analogs (PUFA amines) instead shift *V*_50_ toward more positive voltages (Liin et al., 2015, 2016a; Larsson et al., 2018). This set of experiments implies an electrostatic mechanism of action underlying PUFA effects on *V*_50_, and shows that the magnitude of this electrostatic effect is determined by the protonation status of the PUFA head-group. Notably, we observe clear activating effects by PUFAs on K_V_7.1/KCNE1 (comparable to those on K_V_7.1 alone) during experimental conditions that promote PUFA deprotonation (Liin et al., 2015; Larsson et al., 2018). This suggests that the lack of PUFA effects on K_V_7.1/KCNE1 in our hands is because of KCNE1-induced protonation of the PUFA head-group. Moreover, these findings suggest that provided the head-group of a given PUFA analog has a low pKa value (and thus is deprotonated at physiological pH) said PUFA analog can be utilized to achieve greater effects on K_V_7.1 alone at physiological pH while simultaneously retaining activating effects on K_V_7.1/KCNE1 at physiological pH.

The PUFA head-group is not the sole determinant of the magnitude of PUFA effects. The first indication of this came from studies by Doolan et al. (2002) and Moreno et al. (2015) both of which show DHA induces overall greater effects on K_V_7.1/KCNE1 than EPA does. This is in agreement with our comparison of 16 PUFAs with different tail properties, in which we find that PUFAs with double bonds proximal to the head-group have a higher apparent affinity to K_V_7.1/KCNE1, compared with PUFAs with more distal double bonds (Bohannon et al., 2019). In contrast, there was either no or weak correlation between tail length or number of double tail bonds and K_V_7.1/KCNE1 effects (Bohannon et al., 2019). The proximal localization of double bonds relative to the head-group of DHA may contribute to the relatively greater effect, when compared to EPA at specific concentrations. The mechanistic basis for why proximal double bonds enhance PUFA interaction with K_V_7.1 remains unknown, but may be related to flexibility in the hydrocarbon tail. Studies of PUFA interaction with the Slo1-β1 BK channel suggest that bending at specific carbons distally in the hydrocarbon tail are required for interaction with the channel (Tian et al., 2016). Speculatively, greater bending capabilities close to the head-group may be required for high affinity binding of PUFAs to K_V_7.1.

## Proposed Direct PUFA Sites of Action in K_V_7

Polyunsaturated fatty acid analogs with lower head-group pKa values have been instrumental in determining the mechanism of action of PUFAs on K_V_7.1. PUFA analogs with glycine or taurine head-groups have estimated pKa values that are 1 and 5.5 units lower than that of carboxyl head-groups, respectively, (Bohannon et al., 2020), and are examples of PUFA analogs with exaggerated acute effects on *V*_50_ of both K_V_7.1 and K_V_7.1/KCNE1 at physiological pH (Liin et al., 2015). In experiments utilizing DHA-glycine (DHA-Gly) or AA-taurine (N-AT), we have shown that PUFA analogs interact with S4 to shift *V*_50_. This is observed in voltage-clamp fluorometry experiments that track S4 movement as a PUFA-induced shift in S4 movement toward negative voltages (Liin et al., 2015, 2016b). Individual mutations of the two top arginines in S4 of K_V_7.1 into uncharged glutamines (R228Q and R231Q) either reduce or abrogate entirely the ability of PUFAs to shift *V*_50_ of K_V_7.1 and K_V_7.1/KCNE1 (Liin et al., 2015, 2018). These findings suggest that electrostatic interaction between the negative PUFA head-group and these positively charged arginines in S4 facilitates the upward S4 movement, which contributes to the PUFA effect on *V*_50_ (illustrated as “S4 site” in Figure 1B).

Although charge-neutralizing mutation of S4 arginines removes the *V*_50_ effect of PUFAs, an increase in *G*_max_ of K_V_7.1 and K_V_7.1/KCNE1 is still observed (Liin et al., 2018). This increase in *G*_max_ is also electrostatic in nature, as it is abolished following mutation of a lysine at the top of S6 into an uncharged glutamine (Liin et al., 2018). Experiments combined with molecular dynamics simulations suggest that electrostatic interaction between the negative PUFA head-group and the positively charged lysine at position 326 of S6 (illustrated as “S6 site” in Figure 1B) triggers conformational changes in the ion-conducting pore in and near the selectivity filter. These conformational changes appear to promote potassium ion conductance in a way that contributes to the PUFA effect on *G*_max_. Altogether, these findings suggest that PUFAs have at least two independent sites of electrostatic action in K_V_7.1 channels. Similar sites have been suggested for K_V_7.2 and, to some extent, K_V_7.3 (Larsson et al., 2020).

## Proposed Indirect PUFA Effects on K_V_7

Besides direct effects, PUFAs may influence K_V_7 channels through other mechanisms, including altering bilayer organization and properties or generating active PUFA metabolites. Indication of additional pathways of PUFA modulation of K_V_7 channels comes from prolonged exposure (48 h) of K_V_7.1/KCNE1 channels to DHA or EPA (Moreno et al., 2015). For instance, prolonged DHA or EPA exposure reduces total K_V_7.1 protein levels in COS7 cells, presumably by inducing protein degradation (Moreno et al., 2015). Prolonged exposure also triggers spatial redistribution of K_V_7.1/KCNE1 in the membrane, presumably by disruption of lipid rafts (Moreno et al., 2015). It has been speculated that mechanical properties of the membrane (such as thickness and stiffness) may contribute to altered K_V_7.1/KCNE1 behavior in different membrane microdomains (Moreno et al., 2015). Given that PUFAs regulate other ion channels through modulating the mechanical properties of membranes (Boland and Drzewiecki, 2008), the possible contributions of such indirect PUFA effects on K_V_7 channels in different microdomains warrants further studies.

Regarding the influence of active metabolites, AA is central in oxygenase-driven metabolic pathways that generate leukotrienes and prostaglandins (Shimizu and Wolfe, 1990). Treatment of bullfrog sympathetic neurons with an inhibitor of the lipoxygenase pathway reduces I_M_ and prevents AA-mediated increases in I_M_ amplitude (Villarroel, 1993; Yu, 1995), presumably by impeding downstream AA metabolites that modulate I_M_ effects (Yu, 1995). These findings highlight the multifaceted nature of PUFA signaling and the challenges of dissecting underlying mechanisms in more complex cellular systems and longer time scales.

## Future Directions

Several studies report that micromolar concentrations of PUFAs increase K_V_7.1-7.3 currents and have provided insights into mechanisms of how acute and prolonged PUFA exposure impacts K_V_7 channels. However, there are still several open questions. For instance:

(i) Given the conservation among K_V_7 channels of charged residues identified to be crucial to PUFA effects (Figure 1B), are the effects and underlying mechanisms of action conserved among all K_V_7 isoforms?
(ii) What is the physiological relevance of PUFA effects reported on heterologously expressed K_V_7 channels? PUFA have been reported to alter cardiac and neuronal excitability [e.g., by shortening action potential duration and QT interval, and increasing the threshold for action potential firing (e.g., Leaf et al., 2003; Boland and Drzewiecki, 2008; DeGiorgio and Taha, 2016; Liin et al., 2016a; Skarsfeldt et al., 2020)], and vascular tone [by promoting vascular smooth muscle relaxation (e.g., Hoshi et al., 2013b; Limbu et al., 2018)]. However, as PUFAs act on many types of channels, the extent of Kv7 contribution to such general effects remain to be determined.
(iii) Can a mechanistic understanding of PUFA modulation of K_V_7 channels open up new therapeutic avenues? For instance, how viable is modulation of circulating PUFA levels for the tuning of neuronal and cardiac excitability, and what are the prospects of PUFA analogs as pharmacological K_V_7 channel activators?

To conclude, PUFAs modulate the activity of K_V_7 channels through a range of mechanisms. Studies of heterologously expressed K_V_7 channels have revealed important insights into mechanisms underlying K_V_7 channel activation via direct PUFA-channel interactions. However, several aspects of PUFA modulation of K_V_7 channels remain unclear and require further studies. This is particularly true for comprehending the compounded results of both direct and indirect PUFA effects in differing time scales and cellular systems.

## Author Contributions

All authors designed the study and wrote the manuscript.

## Conflict of Interest

A patent application (#62/032,739) including a description of the interaction of charged lipophilic compounds with the K_V_7.1 channel has been submitted by the University of Miami with SL identified as one of the inventors. The remaining authors declare that the research was conducted in the absence of any commercial or financial relationships that could be construed as a potential conflict of interest.
